# HPV catch-up vaccination of young women: a systematic review and meta-analysis

**DOI:** 10.1186/1471-2458-14-867

**Published:** 2014-08-23

**Authors:** Elisabeth Couto, Ingvil Sæterdal, Lene Kristine Juvet, Marianne Klemp

**Affiliations:** Norwegian Knowledge Center for the Health Services, Health Economic and Drug Unit, St Olavsplass, PO Box 7004, 0130 Oslo, Norway

**Keywords:** Systematic review, Human papillomavirus, Catch-up vaccination

## Abstract

**Background:**

While prophylactic human papilloma virus (HPV) vaccination is considered effective in young girls, it is unclear whether a catch-up vaccination of older girls would be beneficial. We, therefore, aimed to examine the potential health impact of a HPV catch-up vaccination of girls who were too old at the time of vaccine introduction, hence aged 16 and older.

**Methods:**

We systematically searched the literature for randomized clinical trials (RCTs) that examined the effect of HPV vaccines on overall mortality, cancer mortality and incidence, high-grade cervical intraepithelial neoplasia grade 2 and higher (CIN2+), vulvar intraepithelial neoplasia (VIN) and vaginal intraepithelial neoplasia (VaIN) grade 2 and higher lesions (VIN2+ and VaIN2+, respectively) genital warts (condyloma). We considered all lesions and those associated with HPV type(s) included in the vaccines. RCTs reporting on serious adverse events were also eligible. Selected publications were assessed for potential risk of bias, and we ascertained the overall quality of the evidence for each outcome using Grading of Recommendations Assessment, Development and Evaluation (GRADE). Meta-analyses were performed, assuming both random and fixed effects, to estimate risk ratios (RR) and corresponding 95% confidence intervals (CI), using intention-to-treat and per-protocol populations.

**Results:**

We included 46 publications reporting on 13 RCTs. Most of the RCTs had a maximum follow-up period of four years. We identified no RCT reporting on the effect of HPV catch vaccination on overall and cancer related mortality, and on cervical cancer incidence. We found a borderline protective effect of a HPV catch-up vaccination on all CIN2+, with a pooled RR of 0.80 (95% CI: 0.62-1.02) for a follow-up period of 4 years. A HPV catch-up vaccination was associated with a reduction in VIN2+ and VaIN2+ lesions, and condyloma. No difference in risk of serious adverse events was seen in vaccinated participants versus unvaccinated women (pooled RR of 0.99 (0.91-1.08)).

**Conclusions:**

This systematic review indicates that a HPV catch-up vaccination could be beneficial, however the long-term effect of such a vaccination, and its effect on cervical cancer incidence and mortality is still unclear.

**Electronic supplementary material:**

The online version of this article (doi:10.1186/1471-2458-14-867) contains supplementary material, which is available to authorized users.

## Background

Human papillomavirus (HPV) is considered the most common sexually transmitted agent worldwide [1], and most sexually active women and men will experience an HPV infection during their lifetime [2]. More than 100 types of HPV have been identified [3, 4]. However, a small number of HPV types contribute to a large proportion of HPV-related diseases. Most HPV infections resolve within 1-2 years [4], but some are persistent and are recognised as a necessary cause of cervical cancer and for its precursor lesions [4, 5]. Approximately 70% of cervical cancers in the world are attributed to two of the most common HPV types, 16 and 18 [4, 6, 7]. The World Health Organisation (WHO) International Agency for Research on Cancer judged that there was sufficient evidence to support a causal role of HPV 16 infection in carcinoma of the cervix, vulva, vagina, penis, anus, oral cavity, and oropharynx and tonsil [8]. The evidence was also judged sufficient to recognize a causal role of HPV types 18, 31, 33, 35, 39, 45, 51, 52, 56, 58, and 59 in cervical cancer [8]. It was estimated that 5.2% of all cancers worldwide are attributed to HPV infections [7]. Genital warts have been linked to HPV infection [9], with approximately 100% of genital warts (condyloma acuminate) caused by either HPV 6 or 11 [10]. An increasing incidence of genital warts has been described over recent decades in Europe [11].

Efficient prophylactic vaccines could have an important public health impact [12]. Since 2006, two vaccines (Cervarix and Gardasil) have been licensed for girls aged 9 to 26, and through age 45 years in some countries [13], and have been introduced in the childhood immunisation programme in many countries for girls aged 9 to 18 [14]. While prophylactic HPV vaccination has been shown to be effective in young girls, it is still unclear whether a catch-up vaccination of girls who were too old at time of vaccine introduction would be beneficial.

To the best of our knowledge, three meta-analyses have been published to date and they reported results on the effect of HPV vaccination of older girls on persistent HPV infection [15], and only on outcomes associated with the HPV types included in HPV vaccines [16, 17]. In this systematic review, we present results on prevention of all lesions regardless of the HPV status of the lesions. This is an appropriate measure of the public health impact of a HPV catch-up vaccination, as it estimates more accurately the expected reduction in total disease burden after implementation of such a vaccination program.

In this article, we present a systematic review of the international literature to investigate the health impact of a HPV catch-up vaccination of girls who were too old at the time of vaccine introduction. Taking into account the age range covered by HPV vaccines licensing, and the age at introduction of HPV vaccination in different countries, we have therefore examined the health impact of HPV vaccination of girls aged 16 and older.

## Methods

### Eligibility criteria

We included randomised clinical trials (RCT) that examined the efficacy of a HPV catch-up vaccination of young women aged 16 and older. Eligible RCTs examined the effect of HPV vaccines on overall mortality, cancer related mortality, cervical cancer, high-grade cervical intraepithelial neoplasia grades 2 and higher (CIN2+), vulvar intraepithelial neoplasia (VIN) and vaginal intraepithelial neoplasia (VaIN) grade 2 and higher lesions (VIN2+ and VaIN2+, respectively), and genital warts (condyloma). RCTs investigating HPV vaccination safety and reporting on serious adverse events were also eligible. RCTs that used the following comparison groups were included: HPV vaccine against placebo, HPV vaccine against placebo with in addition another vaccine (such as hepatitis B vaccine) used in the intervention and in the placebo groups, or RCTs comparing two different HPV vaccines. No language restriction was applied during the literature search.

### The literature search

We systematically searched several databases from 1999 up to October 2012 (Ovid MEDLINE(R) In-Process & Other Non-Indexed Citations, Ovid MEDLINE(R), Embase, Cochrane Central Register of Controlled Trials, ISI web of Science, PubMed, and Google scholar). Details of the search strategy are provided in the Additional file 1: Appendix. Furthermore, we contacted the pharmaceutical companies with marketing authorization for HPV vaccines in Norway (GlaxoSmithKline AS and Sanofi Pasteur MSD) to obtain additional relevant information. The search was supplemented with papers found in bibliographies of selected articles. We used a search filter to select only RCTs.

### Examined outcomes

To assess the potential health impact of a HPV catch-up vaccination, we have examined several outcomes. We planned to investigate the effect of a HPV catch-up vaccination on overall and cancer mortality, and on cervical cancer incidence. Furthermore, we aimed to examine several female genital HPV related diseases and investigate the association between HPV vaccination and these outcomes. These outcomes were CIN2+, VIN2+, VaIN2+ and genital warts (condyloma acuminata). We also examined the association between HPV vaccination and these lesions considering only HPV related lesions (i.e. HPV type(s) found in the lesion is/are the HPV type(s) covered by the examined vaccine).

We examined also adverse events possibly linked to HPV vaccination. We considered only adverse events reported as serious adverse events in the included publications.

### Data extraction and quality assessment

The selection of articles was carried out by two of the review authors (divided among EC, LJ and IS). All titles and abstracts from the reference lists of articles were screened, and full-text articles were retrieved for publications judged potentially relevant. Each full-length article was assessed for possible inclusion according to the predefined eligibility criteria. Potential disagreements were resolved by discussion with a review author.

Selected publications were assessed for potential risk of bias according to the Cochrane risk of bias tool [18]. All assessments were performed and agreed upon by two of the review authors. No studies were excluded on the basis of high risk of bias.

One review author extracted the data and another verified the information. When data were reported in several publications, we used the publication with the longest follow-up period. If a publication included several trials, preference was given to the publication that included the most trials. We extracted detailed data on characteristics of the clinical trial and participants, on administration of vaccine and control, and on relevant outcomes.

Two review authors assessed the overall quality of the evidence for each selected outcome using GRADE (Grading of Recommendations Assessment, Development and Evaluation) [19].

### Statistical analyses

When possible, we carried out meta-analyses to investigate the association between HPV vaccination and outcomes described above, comparing vaccine and control groups. Extracted data were pooled together by performing meta-analyses using the Review Manager software (RevMan). When the outcome data could not be pooled in meta-analyses, we described the results in a narrative manner.

Random and fixed effect models were used to calculate pooled risk ratios (RR) and corresponding 95% confidence intervals (CI). If we identified fewer than three studies reporting data on the same clinical outcome, we report pooled estimates using fixed effect models. Otherwise, pooled estimates obtained with random effect models are presented.

We performed intention-to-treat (ITT) analyses. However, none of the included studies used an intention-to-treat population including only randomized subjects. We, therefore, used the modified intention-to-treat populations as defined in the included publications. The modified ITT population was most commonly defined as participants who received at least one vaccine or control dose and had at least one follow-up visit post-dose 1. When possible, we conducted also analyses according to per-protocol-population (PPP). The PPP typically included participants who received the three vaccine or control doses. We pooled results published for the safety population, using estimates reported for the longest follow-up period in each study. In most trials, the safety population was similar to the modified ITT population.

## Results

### Literature search and characteristics of included studies

**Figure 1:**
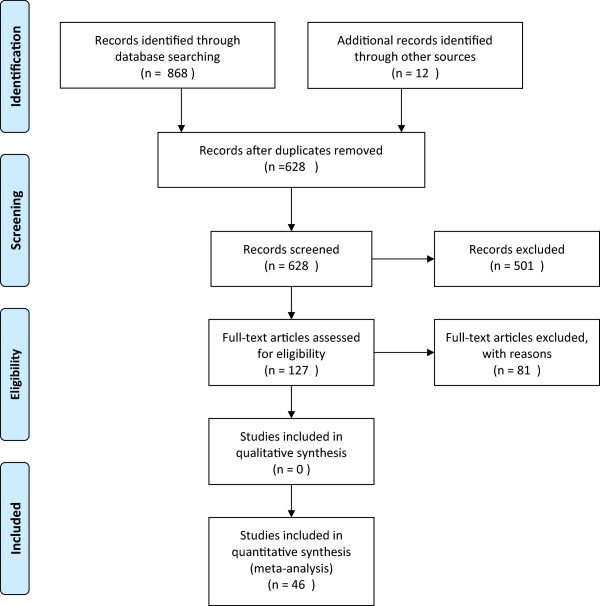
**Selection process of randomized control trials.**

The study selection process is presented in Figure 1. The literature search retrieved 616 references. In addition, we received 12 references from the pharmaceutical companies with marketing authorization for HPV vaccines in Norway. 46 publications, reporting on 13 different RCTs, were selected.

Characteristics from included studies [20–40] are presented in Table 1. Totally, the studies included nearly 40 000 participants. They were conducted in North America (USA and Canada), South America, Europe and Asia. Most clinical trials had a maximum follow-up period of 4 years, with two reporting results after a follow-up of 6 [36], and 8 years [22]. The participants were healthy and non-pregnant women aged 15 to 45 years of age. One of the studies included women aged 9 to 23 years, but the mean age was 17 years [41]. The FUTURE (protocol 19) trial included women aged 24 to 45 (mean age 34 years) [29, 30]. However, we included this study since one of our inclusion criterion was women aged 16 and older. Some studies included only participants with no history of HPV infection and negative HPV tests at entry into the study [34], but most studies recruited participants with fewer than four to six lifetime sex partners [21, 34, 41, 42].

**Table 1:** **Characteristics of randomised control trials included in the review**

Trial [reference]	FUTURE (protocol 5) [20–23]	FUTURE (protocol 7) [23] [24] [26] [39]	FUTURE I (protocol 13) [23] [25–28]	FUTURE II (protocol 15) [23] [25–27]	FUTURE II (protocol 19) [29] [30]	PATRICIA [31–33] [33] [40]	Harper [34–38]
**Phase**	IIa	II	III	III		III	III
**Age range**	16-25 years	16-23 years	16-24 years	15-26 years	24-45 years	15-25 years	15-25 years
**Countries included**	USA	5 countries	16 countries	13 countries	38 international study sites	14 countries	North America and Brazil
**Period of enrollment**	October 1998 to November 1999	2002-2007	January 2002 – March 2003	June 2002 - May 2003	June 2004 - April 2005	May 2004 -June 2005	November 2003 – July 2004
**Inclusion criteria**	Not pregnant, no prior Pap tests and with a lifetime history of 0-5 male sex partners	Non pregnant, healthy women, no prior abnormal Pap smears, and with a lifetime history of 0-4 male sex partners. Among virgins, enrolment was limited to those 18 years and over of age and seeking contraception.	Not pregnant, reporting no prior Pap tests and lifetime history of 0-4 sex partners. No history of genital warts	Not pregnant, reporting no prior Pap tests and lifetime history of 0-4 sex partners.	Healthy non pregnant women, agreed to contraception, intact cervix, with no history of coloscopy.	Healthy non pregnant women, 0-6 sexual partners, agreed to contraception, intact cervix, with no history of coloscopy. Not breastfeeding and without chronic or autoimmune disease	Healthy women with 0-6 sexual partners. No history of abnormal Pap test or ablative or extensional treatment for external condylomata; who were cytologically negative, seronegative for HPV-16 and HPV-18 antibodies by ELISA, and HPV-DNA negative by PCR for 14 high risk HPV types, no more than 90 days before study entry.
**Intervention comparator**							
**Vaccine**	HPV 16 vaccine (N = 768)	HPV-6/11/16/18 (N = 276)	HPV-6/11/16/18 (N = 2723)	HPV-6/11/16/18 (N = 6087)	HPV 6, 11, 16, 18 (N = 1911)	HPV16/18 (N = 8093)	HPV 16/18 (N = 560)
**Comparator**	Placebo (N = 765)	Placebo (N = 275)	Placebo (N = 2732)	Placebo (N = 6080)	Placebo (N = 1908)	Hepatitt A vaccine (N = 8069)	Placebo (N = 553)
**Administration schedule**	day 1, month 2 and month 6.	day 1, month 2 and month 6.	day 1, month 2 and month 6.	day 1, month 2 and month 6.	day 1, month 2 and month 6.	0,1 and 6 month	0,1 and 6 month
**Length of follow up**	Up to 48 months	36 months and extension study of 2 years	Up to 48 months	Up to 48 months	Median 4 years	Up to 48 months	incl 27 mths and 4,5 yrs; ≤ 8.4 yrs (Brazilian centers)
**Study populations**							
**Intention to treat (ITT)**	Subjects who received at least one vaccination, included all protocol violators and subjects who tested positive for HPV-16 infection at enrollment.	Subjects who were naıve to the relevant HPV type(s) at enrolment and had received at least one vaccination	Subjects who received at least 1 dose of vaccine or placebo and returned for follow-up.	Subjects who received at least 1 dose of vaccine or placebo and returned for follow-up.	Subjects who received X1 dose of vaccine or placebo and returned for follow-up.	Total vacine cohort (TVC) included all who received at least one vaccine dose and were evaluable for efficacy, irrespctive of baseline HPV status, cytological status, and serostatus.	Subjects who had received at least one dose of study vaccine or placebo in the initial efficacy study, and who had any data available for outcome measurement in the extended follow-up phase.
**Per protocol population (PPP)**	Subjects who tested seronegative for HPV16 at the first study visit, tested negative for HPV16 DNA at all visits between day 1 and month 7 inclusive, and completed the entire three dose vaccine series.	Subjects who were PCR and seronegative to HPV 6, 11, 16, or 18 at enrolment, remained PCR-negative to the same vaccine-HPVtype (s) (to which they were naı¨ve at enrolment) through 1 month postdose three, received three doses of vaccine or placebo within 1 year, and did not violate the protocol.	Subjects who received all 3 doses of vaccine or placebo within 12 months. Were seronegative and HPV DNA negative on PCR analysis for HPV-6, HPV-11, HPV-16, or HPV-18.	Subjects who received all 3 doses of vaccine or placebo within 12 months. Were seronegative and HPV DNA negative on PCR analysis for HPV-6, HPV-11, HPV-16, or HPV-18.	Subjects who were seronegative at day 1 and PCR-negative (from day 1 through month 7 to the relevant vaccine HPV type(s) and did not violate the protocol.	According to protocol for efficacy (ATP-E) included all participants that received three doses of vaccine or placebo with a negative HPV DNA test, seronegative for HPV16 and/or 18 and with normal or low-grade cytology on day 1.	Subjects in the extended follow up phase who received three doses of HPV 16/18 vaccine or placebo, and who were negative for high-risk HPV DNA and seronegative for HPV 16 and 18 DNA
**Safety population**	Included all randomized participants		Included all randomized participants with follow-up information	Included all subjects who completed the vaccination report card from day 1 through day 15 after	Included all randomized participants with follow-up information	Included all randomized participants	Included all assessible women who did not use any investigational or non-registered product or any HPV vaccine other than study vaccine during the study period.
**Outcomes used in article**	HPV related CIN2+	HPV related CIN2+ VIN2+ /VaIN2+ HPV related Condyloma SAE	CIN2+ HPV related CIN2+ VIN2+ /VaIN2+ related and not Condyloma SAE	CIN2+ HPV related CIN2+ VIN2+ /VaIN2+ related and not Condyloma SAE	CIN2 Condyloma VIN2+ VaIN2+ SAE	CIN2+ HPV related CIN2+ SAE	mortality CIN2+ SAE
**Risk of bias**	None	None	None	None	None	None	None
**Funding source**	Merck Research Laboratories	Merck Research Laboratories	Merck Research Laboratories	Merck Research Laboratories	Merck Research Laboratories	GlaxoSmith Kline Biologicals	GlaxoSmith Kline Biologicals

Vaccines used in the trials were the bivalent vaccine containing HPV 16 and 18 virus-like particles (VLP) from GlaxoSmithKline, or the monovalent vaccine containing HPV 16 VLP and the quadrivalent vaccine containing HPV 6, 11, 16 and 18, both from Sanofi Pasteur MSD. All trials used placebo as comparator except for two studies: one used hepatitis B vaccine in both the intervention and the control groups [43], and one compared the bivalent and the quadrivalent vaccines [44]. All vaccines were given as three doses during a six months period (at day 1, months 2 and 6; or at months 0, 1 and 6).

Some of the included studies had unclear allocation concealment and unclear blinding. However, all studies were assessed as having low risk of bias.

While, one of our aims was to investigate the effect of a HPV catch-up vaccination on overall and cancer related mortality, and on the incidence of cervical cancer, no RCT examining these outcomes were identified.

### Effect of HPV vaccines on outcomes identified in relevant studies

Table 2 summarises the effects of HPV vaccine versus placebo or no vaccine and the quality of evidence for each outcome.

**Table 2:** **Summary of findings table for HPV vaccine versus placebo or no vaccine**

Outcomes	Illustrative comparative risks* (95% CI)		Relative effect (95% CI)	No of Participants (studies)	Quality of the evidence (GRADE)
	Assumed risk	Corresponding risk			
	Placebo, no vaccine or other vaccines	HPV vaccines			
**Cancer mortality** ^**&**^					
**Cervical cancer** ^**&**^					
**CIN 2+ ITT** (any HPV type) (4-year follow-up)	51 per 1000	41 per 1000 (32 to 52)	RR 0.8 (0.62 to 1.02)	39381 (5 studies)	⊕⊕⊕⊝ moderate^1,2^
**CIN2+ PPP** (any HPV type) (4-year follow-up)	29 per 1000	14 per 1000 (6 to 34)	RR 0.49 (0.21 to 1.14)	1096 (1 study)	⊕⊕⊝⊝ low^2,3^
**CIN2+ ITT** (any HPV type) (6-year follow-up)	34 per 1000	10 per 1000 (4 to 27)	RR 0.29 (0.11 to 0.78)	1002 (1 study)	⊕⊕⊕⊝ moderate^2,4^
**CIN2+ ITT** (any HPV type) (8-year follow-up)	85 per 1000	54 per 1000 (23 to 128)	RR 0.64 (0.27 to 1.52)	290 (1 study)	⊕⊕⊝⊝ low^2,5,6^
**CIN2+ lesions ITT** (HPV 16 and/or 18 related) (4- year follow up)	22 per 1000	12 per 1000 (10 to 14)	RR 0.54 (0.44 to 0.67)	42652 (7 studies)	⊕⊕⊕⊕ high^2^
**CIN2+ ITT** (HPV 16 and/or 18 related) (8-year follow-up)	31 per 1000	9 per 1000 (3 to 30)	RR 0.29 (0.09 to 0.96)	721 (2 studies)	⊕⊕⊕⊝ moderate^4,7^
**CIN2+ PPP** (HPV (16 and/or 18 related) (4- year follow up)	11 per 1000	1 per 1000 (0 to 2)	RR 0.05 (0.01 to 0.16)	35023 (6 studies)	⊕⊕⊕⊕ high^2^
**Genital warts ITT** (any HPV type) (4-year follow-up)	40 per 1000	15 per 1000 (13 to 19)	RR 0.38 (0.31 to 0.47)	17391 (2 studies)	⊕⊕⊕⊕ high^2^
**Genital warts ITT** (HPV 6 and/or 11 related) (4-5 year follow up)	30 per 1000	8 per 1000 (4 to 19)	RR 0.28 (0.12 to 0.65)	21686 (4 studies)	⊕⊕⊕⊕ high^2^
**VIN2+ and VaIN2+ ITT** (any HPV type) (4-year follow-up)	7 per 1000	3 per 1000 (2 to 5)	RR 0.49 (0.32 to 0.76)	17391 (2 studies)	⊕⊕⊕⊝ moderate^2,4^
**VIN2+ and VaIN 2+ ITT** (HPV related) (4-5-year follow-up)	4 per 1000	3 per 1000 (0 to 60)	RR 0.72 (0.03 to 15.02)	21694 (4 studies)	⊕⊕⊝⊝ low^1,6^
**Serious Adverse Events** (Follow-up: >7 months^8^, longest reported follow up)	44 per 1000	44 per 1000 (40 to 48)	RR 0.99 (0.91 to 1.08)	43342 (14 studies)	⊕⊕⊕⊝ moderate^2,9^

*The basis for the assumed risk is the median control group risk across studies. The corresponding risk (and its 95% confidence interval) is based on the assumed risk in the comparison group and the relative effect of the intervention (and its 95% CI).

*CI* Confidence interval, *RR* Risk ratio;

GRADE Working Group grades of evidence.

High quality: Further research is very unlikely to change our confidence in the estimate of effect.

Moderate quality: Further research is likely to have an important impact on our confidence in the estimate of effect and may change the estimate.

Low quality: Further research is very likely to have an important impact on our confidence in the estimate of effect and is likely to change the estimate.

Very low quality: We are very uncertain about the estimate.

^&^No studies that reported on overall and cervical cancer mortality, and cervical cancer incidence were identified.

^1^I-square >75%.

^2^Funded by vaccine provider (we did not downgrade).

^3^Few events, high number of loss to follow-up.

^4^Few events.

^5^Participants were not blinded in this extended follow-up study.

^6^Few events and wide confidence interval. Both estimates of relative and absolute effects have wide confidence intervals.

^7^Participants were not blinded in one of the extended follow-up studies.

^8^We used the longest reported follow-up for each trial.

^9^We have reported the results for the safety population as it was defined in each of the studies. Might have led to uncertain loss to follow up. Serious adverse events are defined differently in the studies.

#### Overall mortality

Overall mortality was seldom reported, and primarily only in the text. Overall mortality was reported in 7 RCTs [28, 29, 32, 34, 41, 45]. The authors reported that none of the recorded deaths were considered to be related to the intervention in the vaccine or control groups.

#### CIN2+

**Figure 2:**
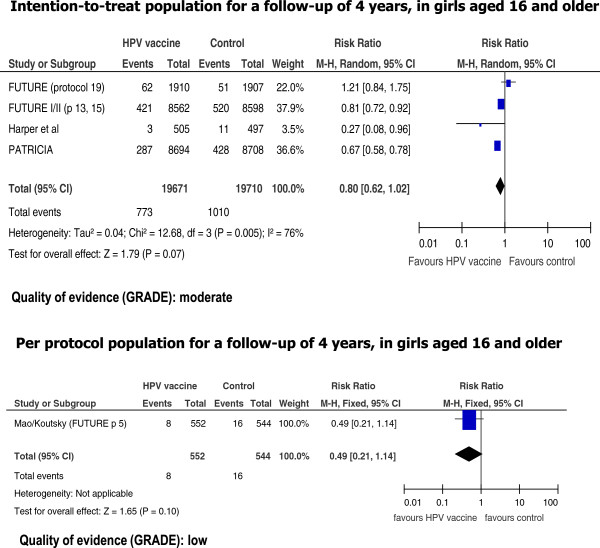
**Risk of cervical intraepithelial neoplasia grade 2 and higher lesions associated with HPV vaccination.**

The intention-to-treat (ITT) analysis based on five studies showed a borderline statistically significant reduction in CIN2+ lesions associated with HPV vaccination with a pooled RR of 0.80 (95% CI: 0.62-1.02) for a follow-up period of 4 years (Figure 2). The quality of the evidence was judged moderate. Two of the included studies published results for longer follow-up periods, and reported RRs of 0.29 (0.11-0.78) [36], and 0.64 (0.27-1.52) [22] for follow-up of 6 and 8 years, respectively (results not shown).

The reported RR using the per protocol population (PPP) showed a non statistically significant reduction for all CIN2+ lesions after a four year follow-up period (RR: 0.49; 95% CI: 0.21-1.14) (Figure 2). However, this finding was based on only one RCT, and the quality of the evidence was considered low.

**Figure 3:**
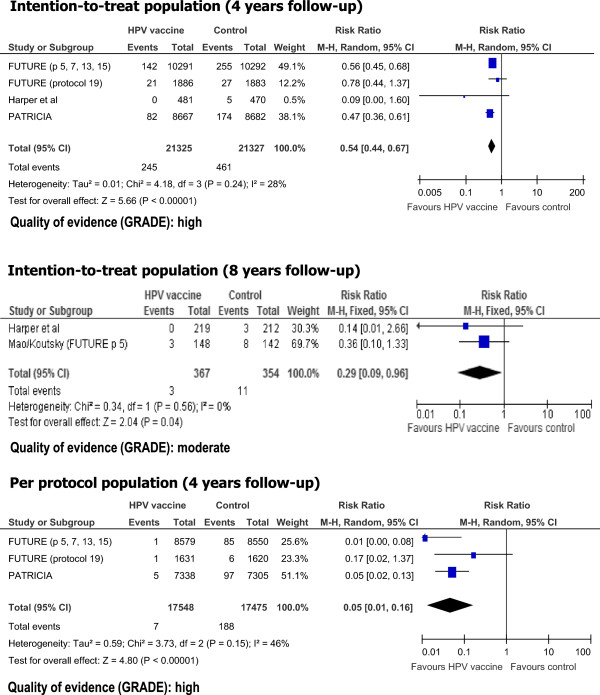
**Risk of HPV related cervical intraepithelial neoplasia grade 2 and higher lesions associated with HPV vaccination.**

When considering only CIN2+ HPV related lesions, we found statistically significant reductions in risk with HPV vaccination both for studies using ITT and PPP populations (Figure 3). The pooled RRs were 0.54 (0.44-0.67) for the ITT population, and 0.05 (0.01-0.16) for the PPP population, both for a 4 years follow-up period. The quality of the evidence was considered high for both estimates. Two studies reported data for 721 participants from the ITT population for 8 years follow up (Figure 3). The pooled RR was 0.29 (0.09-0.96). The quality of the evidence for this outcome was considered moderate.

#### VIN2+, VaIN2+

**Figure 4:**
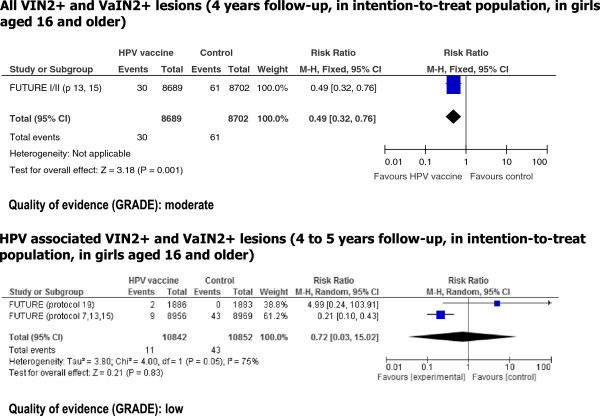
**Risk of vulvar and vaginal intraepithelial neoplasia lesions associated with HPV vaccination.** Legend: VIN2+: vulvar and vaginal intraepithelial neoplasia grade 2 and higher. VaIN2+: vaginal intraepithelial neoplasia grade 2 and higher

We found a statistically significant reduction in risk of all VIN2+ or VaIN2+ lesions with HPV vaccination (RR = 0.49; 95% CI = 0.32-0.76) based on two RCTs reported in one publication (Figure 4). The quality of the evidence for this outcome was considered moderate. However, when considering only published estimates on HPV related VIN2+ or VaIN2+ (from four studies), the reduction in risk was not statistically significant (pooled RR = 0.72; 0.03-15.02) (Figure 4). The quality of the evidence for this outcome was low.

#### Condyloma acuminata

**Figure 5:**
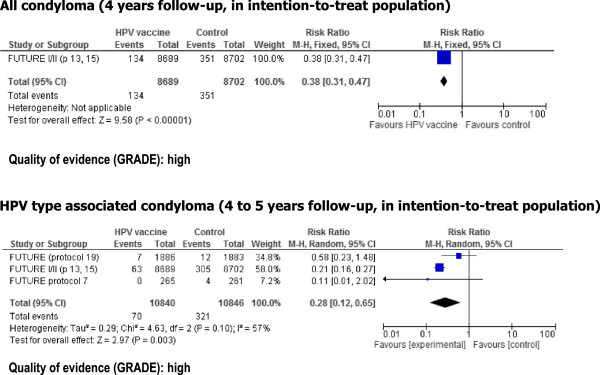
**Risk of condyloma associated associated with HPV vaccination in girls aged 16 and older.**

HPV vaccination was associated with a reduction in risk of condyloma, both for all condyloma and for those related to HPV types included in HPV vaccines in the ITT population (Figure 5). The reported RR, based on two RCTs, was 0.38 (0.31- 0.47) for all condyloma, and the pooled RR was 0.28 (0.12-0.65) for HPV related condyloma. The quality of the evidence for these two outcomes was high.

#### Serious adverse events

**Figure 6:**
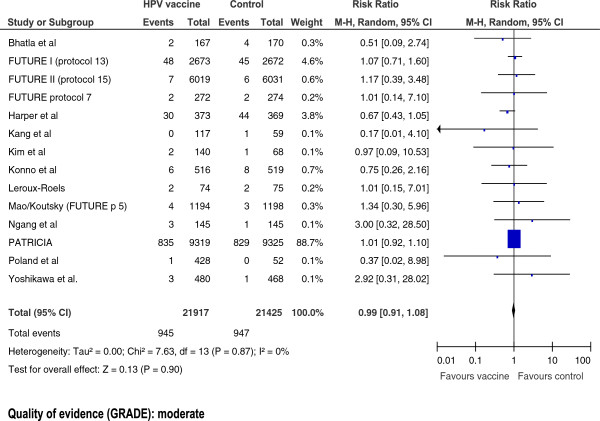
**Risk of serious adverse events associated with HPV vaccination in girls aged 16 and older.**

We included 14 studies that reported estimates of the association between HPV vaccination and serious adverse events. The risk of having a serious adverse event was similar in both the vaccine and control groups (Figure 6). The pooled RR was 0.99 (0.91-1.08). The quality of the evidence for this outcome was moderate.

We identified one publication that compared the bivalent vaccine with the quadrivalent vaccine, and examined possible differences safety between these two vaccines [44]. However, the quality of the evidence was judged low, and no statistically significant difference was found (RR = 1.05; 95% CI: 0.59-1.05) (results not shown).

## Discussion

This systematic review shows that there is a protective effect of HPV vaccination against CIN2+ lesions associated with the HPV types included in HPV vaccines, all VIN2+ and VaIN2+, and condyloma acuminate (HPV related and not). The results also indicate a protective effect against all CIN2 lesions (HPV associated and not). No difference in the occurrence of serious adverse events was found in vaccinated participants compared to control groups.

High grade cervical lesions (CIN2+) were proposed by the WHO as an appropriate surrogate outcome for RCTs examining the effect of HPV vaccination [46]. A reason for this was that since screening for cervical cancer is available, conducting RCTs considering cervical cancer as main outcome would be unethical. CIN lesions are precursors of invasive cervical cancer and while most lower grade CIN (i.e. CIN1) regress spontaneously to normal, a higher percentage of CIN2+ become malignant making these lesions a more appropriate outcome to examine [47]. However, when investigating HPV vaccination efficacy, the main outcome of interest remains cervical cancer, and CIN2+ lesions are mainly examined to extrapolate on the possible effect of HPV vaccination on cervical cancer. One should be cautious when interpreting results on CIN2+ lesions on the possible effect of HPV vaccination on cervical cancer risk. For example, differing results for cervical cancer than those published up to date on precursor lesions could be expected if the HPV types involved in precursor lesions were different to those related to cervical cancer. A meta-analysis reported that while HPV 16 and 18 are the two most common types both in high-grade squamous intraepithelial lesions (HSIL) and squamous cell carcinoma of the cervix (SCC), these HPV types were reported to be more common in SCC than in HSIL with prevalence ratios of 1.21 (95% CI: 1.16-1.26), and 1.79 (1.56-2.10) for HPV 16 and 18, respectively [48]. It is, therefore, possible that higher protective effect of HPV vaccination would be found for HPV related cervical cancers compared to precursor lesions.

HPV vaccines were shown to be highly efficacious to prevent persistent infections with HPV [16, 17, 49, 50]. Previous meta-analyses have presented results only for persistent HPV infection, and pre-cancerous lesions or condyloma associated with HPV types covered by the vaccines [16, 17]. To the best of our knowledge, our systematic review is the first to present the effect of HPV catch-up vaccination on all pre-cancerous lesions and condyloma, considering both all lesions and those associated with particular HPV types. Examining the effect of HPV vaccination on relevant outcomes regardless of these being related to any HPV type enables a more accurate estimate of the total disease reduction that could be expected after vaccination. We found a borderline statistical significant protection of a HPV catch-up vaccination on all CIN2+ lesions. While 4 out of 5 included RCTs found a protective effect of HPV vaccination on all CIN2+ lesions, one trial reported a non statistically significant increased risk associated with HPV vaccination (RR: 1.21, 95% CI: 0.84-1.75). Participants of this study were older than those from other studies (age range: 24 to 45, mean age 34 years), and somewhat older than women who would most commonly be targeted by a catch-up vaccination. Observational studies examining early vaccine impact have shown a decline in high-grade cervical lesions [51].

While HPV types 16 and 18 are the two most common HPV types worldwide, prevalence of HPV types differs geographically [1]. In included trials, participants are from different geographical regions. They are for example, from Northern America where HPV 16 and 53 are the two most common HPV types, or from Southern America were HPV16 and 58 are the most prevalent types [1]. When ascertaining HPV vaccination efficacy using CIN2+ regardless of the lesions being related to HPV types, differing results may be found according to how common the HPV types included in the vaccine(s) are in the geographical area of interest. In regions were the most common HPV types are included in the vaccine, like in Northern Europe where HPV 16 and 18 are the two most common types, one could expect a stronger protective effect of HPV vaccination on all CIN2+ lesions than in areas where other HPV types are more frequent.

While prophylactic HPV vaccination is considered effective and cost-effective in young girls, it is unclear whether a catch-up vaccination of older girls would have beneficial effect on health outcomes. This is primarily due to differences of HPV status between young and older girls, with younger more likely to be free of HPV infection. In the population included in this systematic review, the HPV status of participants varies, because of the age-range examined in this review, and differences in studies inclusion criteria: Some studies included only participants with no history of HPV infection and negative HPV tests at entry into the study [34], and other studies recruited participants with fewer than four to six lifetime sex partners [21, 34, 41, 42]. The population considered in this systematic review is, therefore, representative of a population targeted by a HPV catch-up vaccination that would be composed of girls both HPV naïve and not. Possible differences in HPV vaccination of young women compared to older women could, also, be explained by potential differences in HV distribution by age [52]. Brotherton et al. reported that HPV type 16 was more prevalent among younger women [52].

The conducted RCTs on the efficacy of HPV vaccination have a relatively short follow-up period with most results published up to date based on follow-up periods of approximately four years. Two included trials published results after longer follow-up periods (6 and 8 years) but the results were based on few participants and are inconclusive [22, 36]. The evidence published so far does not allow us to conclude on the long term effect of HPV vaccination. Furthermore, as cancer takes a long time to develop, longer follow-up periods are required to ascertain the efficacy of HPV vaccination in preventing cervical cancer. The use of population-based registries was described as the best study design to answer this question, and preliminary observations suggested that results on vaccine efficacy against cervical cancer could be available within the next 5 to 10 years [53]. Furthermore, due to lack of long follow-up periods of RCTs published to date, the durability of immune response to HPV vaccines is unknown. Further investigation is, therefore, needed to examine whether booster vaccination(s) would be required after initial vaccination.

In most developed countries, national cervical cancer screening programs have been implemented, with a reported reduction in cervical cancer incidence probably partly due to these programs [54–56]. A public health policy aiming at implementing a HPV vaccination should be done considering the interconnection between the HPV vaccination program and the cervical cancer screening program. While HPV vaccination could present a valuable primary prevention for cervical cancer and other HPV related diseases, certain gaps need to be addressed. Although cross-protection against other HPV types than those covered by the vaccines is possible [57], the two vaccines used nowadays do not protect against all HPV types. HPV type replacement following HPV vaccination has also been a discussed issue [58, 59]. However, up to date, it is not clear, whether there will be an increase of HPV types that have also been linked to cancer but not present in HPV vaccines [60]. Lower coverage could be possible for a catch-up vaccination strategy compared to vaccination of the primary target population of young girls recruited, for example, through schools (i.e. 9-13 years old). Finally, while long term efficacy of HPV vaccines is unproven to date, such an efficacy is required to prevent cervical cancer. A good secondary prevention, such as cervical cancer screening programs, could cover these potential gaps in HPV vaccination. However, concerns have been raised on the possible future consequences of HPV vaccination on the attendance at the cervical cancer screening programs, with possible lower compliance among vaccinated women who perceived themselves at lower risk of cervical cancer [61, 62]. Furthermore, since this systematic review indicates a protective effect of HPV vaccination on all CIN2+ lesions, we could therefore foresee a decrease in screening referrals and subsequent treatments of pre-malignant lesions in the future. The current evidence points towards the need of coordinated policies for HPV vaccination and cervical cancer screening [63, 64].

The evidence, to date, shows that a HPV catch-up vaccination can be considered safe, with no differences seen in serious complications between the vaccination and the control groups. However, the number of cases of published clinical studies may not be sufficient to determine the occurrence of rarely occurring (severe) adverse events in a reliable way. Furthermore, due to the relatively short follow-up period since HPV vaccination implementation in different countries, the long-term safety of the vaccines is unclear and needs to be further monitored in future studies. While different adverse events have been reported, as we examined only events reported as serious this ensures a better homogeneity of the ascertained outcome across studies.

## Conclusions

This systematic review indicates that HVP catch-up vaccination could be a valuable primary prevention against cervical cancer and national catch up vaccination programs have already been implemented in several countries. However, the long term effect of such a vaccination strategy and its impact on cervical cancer prevention remains to be determined.

## Electronic supplementary material

Additional file 1: **Appendix.** (DOCX 14 KB)

## Abbreviations

HPV: Human papilloma virus
RCT: Randomized clinical trial
CIN: Cervical intraepithelial neoplasia
CIN2+: Cervical intraepithelial neoplasia grade 2 and higher
VIN: Vulvar intraepithelial neoplasia
VIN2+: Vulvar intraepithelial neoplasia grade 2 and higher
VaiN: Vaginal intraepithelial neoplasia
VaIN2+: Vaginal intraepithelial neoplasia grade 2 and higher
GRADE: Grading of Recommendations Assessment, Development and Evaluation
RR: Risk ratio
CI: Confidence interval
ITT: Intention-to-treat
PPP: Per-protocol-population
WHO: World Health Organisation.
