# Supplementary material for: HPV catch-up vaccination of young women: a systematic review and meta-analysis
Source: BMC Public Health. 2014 Aug 23;14:867. doi: 10.1186/1471-2458-14-867 (PMC4159543; doi:10.1186/1471-2458-14-867)
Supplement: Supplementary file 1 — Additional file 1: Appendix. (DOCX 14 KB) [file 12889_2014_7005_MOESM1_ESM.docx]

Appendix

Databases: Embase, Ovid Medline, Cochrane Library; Central, ISI web of Science, PubMed, Clinical Trials.gov, WHO ICTRP, Google scholar.

Study design: RCT; search filter based on Ovid’s filter “Therapy Maximizes specificity”, extended with “random*.tw”.

Time limit: 1999 - 2012.

Result: 615 RCT (868 including dupl.).

Searched by: Ingrid Harboe, research librarian.

Example of search strategy:

Database: Embase 1980 to 2012 Week 38, Ovid MEDLINE(R) In-Process & Other Non-Indexed Citations and Ovid MEDLINE(R) 1946 to Present.

Date: 04.10.2012.

Result: 448 RCT.

| # | Searches | Results |
| --- | --- | --- |
| 1 | Papillomavirus infections/ use prmz | 13426 |
| 2 | Papillomavirus infections/ use emez | 2854 |
| 3 | Papillomaviridae/ use prmz | 18154 |
| 4 | Papilloma virus/ use emez | 9369 |
| 5 | Warts/ use prmz | 3806 |
| 6 | Wart virus/ use emez [Underordnet emneord for Papilloma virus/] | 21446 |
| 7 | Condylomata acuminata/ [U e for Wart virus] | 10074 |
| 8 | Human papillomavirus 6/ use prmz | 252 |
| 9 | Human papillomavirus type 6/ use emez | 1121 |
| 10 | Human papillomavirus 11/ use prmz | 232 |
| 11 | Human papillomavirus type 11/ use emez | 1026 |
| 12 | Human papillomavirus 16/ use prmz | 2127 |
| 13 | Human papillomavirus type 16/ use emez | 5375 |
| 14 | Human papillomavirus 18/ use prmz | 891 |
| 15 | Human papillomavirus type 18/ use emez | 2782 |
| 16 | papillomavir*.tw. [= -virus/ -viridae] | 48019 |
| 17 | papilloma vir*.tw. | 8898 |
| 18 | hpv*.tw. | 51345 |
| 19 | wart virus*.tw. | 257 |
| 20 | condylomata acuminat*.tw. | 2151 |
| 21 | genital wart*.tw. | 3684 |
| 22 | venereal wart*.tw. | 145 |
| 23 | or/1-22 | 87192 |
| 24 | Papillomavirus Vaccines/ use prmz [=human papilloma virus vaccines i Medline] | 3229 |
| 25 | Viral Vaccines/ use prmz | 18904 |
| 26 | Wart virus vaccine/ use emez [=hpv vaksine i Embase] | 5437 |
| 27 | Virus vaccine/ use emez | 16768 |
| 28 | Cancer vaccines/ use prmz | 9149 |
| 29 | Cancer vaccine/ use emez | 9689 |
| 30 | *Vaccines/ use prmz | 10142 |
| 31 | *Vaccine/ use emez | 17399 |
| 32 | vaccin*.tw. | 421906 |
| 33 | Immunization/ | 112477 |
| 34 | (immuni?e or immuni?ation*).tw. | 165835 |
| 35 | or/24-34 | 570950 |
| 36 | 23 and 35 | 14897 |
| 37 | Animals/ or Animal/ or Animal Experiment/ | 8367690 |
| 38 | Humans/ | 26303234 |
| 39 | 37 not (37 and 38) | 6438647 |
| 40 | 36 not 39 [resultat uten animals] | 13742 |
| 41 | limit 40 to yr = “1999 -Current” | 12793 |
| 42 | Randomized Controlled Trial.pt. | 337758 |
| 43 | Randomized Controlled Trial/ | 667268 |
| 44 | random*.tw. | 1372370 |
| 45 | or/42-44 | 1549338 |
| 46 | 41 and 45 | 863 |
| 47 | remove duplicates from 46 [RCT] | 530 |
| 48 | 47 use emez [RCT] | 480 |
| 49 | limit 48 to embase | 398 |
| 50 | 47 use prmz [RCT] | 50 |
